# Left Atrial Giant Cell Myocarditis Presenting as a Tumor: First-in-Man Case Report

**DOI:** 10.21470/1678-9741-2017-0242

**Published:** 2018

**Authors:** Omer Tanyeli, Yuksel Dereli, Niyazi Gormus, Mustafa Cihat Avunduk

**Affiliations:** 1 Department of Cardiovascular Surgery, Meram Medicine Faculty, Necmettin Erbakan University, Konya, Turkey.; 2 Department of Pathology, Meram Medicine Faculty, Necmettin Erbakan University, Konya, Turkey.

**Keywords:** Myocarditis, Heart Failure, Heart Neoplasms

## Abstract

Giant cell myocarditis is a rare and highly lethal disorder with resultant cardiac insufficiency. It necessitates aggressive immune suppression therapy, although the results are often fatal. When it affects only the atria, the characteristics of the disease changes completely. In this case report, we present atypical presentation of atrial giant cell myocarditis with mass lesion, which completely resolved after successful surgical resection without immuno suppression therapy.

**Table:** 

Abbreviations, acronyms & symbols	
BMS	= Bare-metal stent
CS	= Cardiac sarcoidosis
DES	= Drug-eluting stents
GCM	= Giant cell myocarditis
ICU	= Intensive care unit
LA	= Left atrium
LVEF	= Left ventricular ejection fraction
PTCA	= Percutaneous transluminal coronary angioplasty
RCA	= Right coronary artery
RF	= Rheumatic fever

## INTRODUCTION

Giant cell myocarditis (GCM) is a rare and highly lethal disorder with resultant cardiac insufficiency. It requires aggressive immune suppression therapy, although the results are often fatal. Recently, a new entity of GCM was described. In this form of the disease, when GCM affects only the atria, the characteristics of the disease changes completely^[^^1^^]^. In this case, we present first-in-man successful treatment of a 53-year-old male patient with the diagnosis of atrial GCM, presenting in the form of a tumor, and his status at the end of 24 months follow-up.

## CASE REPORT

**Fig. 1:**
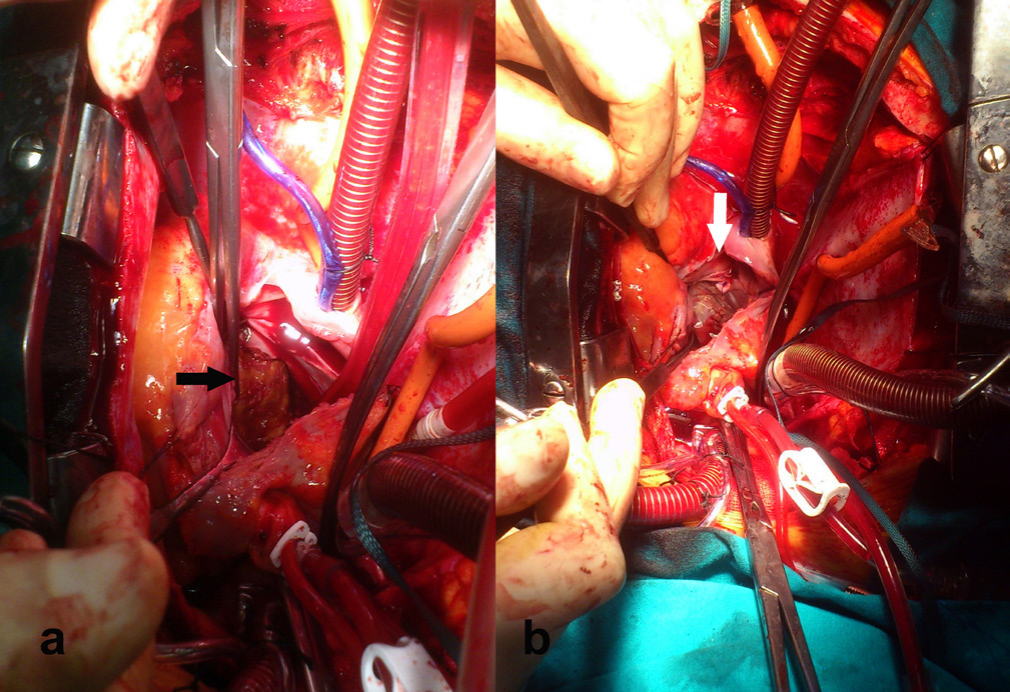
Intraoperative view of atrial giant cell myocarditis, which invaded the myocardium towards the hypertrophied posterior wall of the left atrium (A) and defective posterior atrial wall repaired and strengthened by autologous pericardium, fixed by 0.625% glutaraldehyde solution (B).

A 53-year-old man, who had non-exertional chest pain with positive exercise stress test, was hospitalized by the cardiology department. Total occlusion of the right coronary artery (RCA) was detected in the cath-lab. After performing percutaneous transluminal coronary angioplasty (PTCA), long segment drug-eluting stents (DES) and bare-metal stent (BMS) were placed. After the procedure he became hypotensive. In order to exclude pericardial effusion, transthoracic echocardiography was performed. In the left atrium (LA), a 6,5x4 cm mass was detected with LA dilatation and estimated systolic pulmonary artery pressure over tricuspid regurgitant jet was 43 mmHg. Then, he was sent to our clinic for surgery with possible diagnosis of LA myxoma. His left ventricular ejection fraction (LVEF) was 60%. We decided to perform surgical excision of the mass. Following median sternotomy, we reached the LA via transseptal approach. We saw a giant mass in the LA, nearby the mitral valve. The mitral valve was intact without any deformation. We excised the mass, which invaded the myocardium towards the posterior wall of the LA. The LA was hypertrophic in nature, so all the mass with LA was resected. The mass consisted of central necrotic parts which did not resemble to atrial myxoma. Intraoperative view of the mass is shown in Figure 1A. The defective posterior atrial wall was then repaired by autologous pericardium, fixed by 0.625% glutaraldehyde solution (Figure 1B). Intraoperative frozen pathology specimen was reported as benign tissue, rich in fibrosis, but not myxoma. After closing the septum and the right atriotomy, RCA bypass over PD segment was performed using saphenous vein graft. The aortic occlusion time was 141 minutes. Since the patient had first-degree heart blockage, a temporary pacemaker lead was inserted and the operation was completed in routine manner. Postoperative period was uneventful. He had sinus rhythm after 24 hours, stayed in intensive care unit (ICU) for 2 days, and discharged from the hospital at postoperative 8^th^ day.

**Fig. 2:**
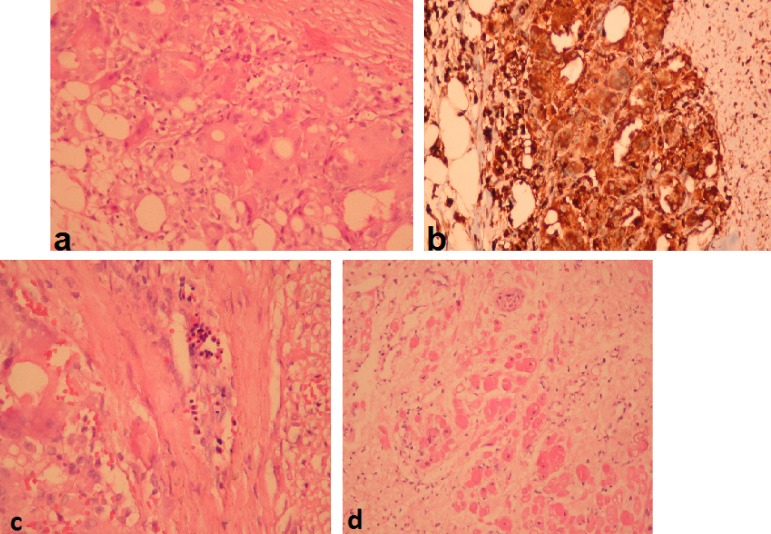
Pathologic specimen prepared by hematoxylin eosin (x20; figure 2A) and immunohistochemical CD 68 staining (x20; figure 2B), showing necrotic changes with fibrous tissue and multinucleated giant cells. Multicellular giant cells and inflammatory cell infiltration (figure 2C, with hematoxylin-eosin, x20) and lymphocytes, histiocytes and infrequent eosinophils in the muscle fibers (figure 2D, with hematoxylin-eosin, x20) are shown in the figure.

Histopathologic examination of the specimen revealed necrotic areas, lymphocytic and histiocytic infiltration and sporadic eosinophilia in the striated muscle fibers. There were also multinuclear giant cells. These giant cells showed positive expression with CD45 and CD68, with the final diagnosis of GCM (Figure 2A-D). After the accurate diagnosis of GCM, he was followed-up by echocardiography in the 10^th^ day, 1^st^ month, and every 3 months thereafter. He clinically improved after the operation, and still had LVEF of 55% at the end of 24 months follow-ups. He took only acetylsalicylic acid (100 mg/day), ramipril (5 mg/day) and bisoprolol (5 mg/day) treatment after the operation without immune suppression therapy.

## DISCUSSION

Idiopathic GCM is a rare form of fulminant myocarditis which has a poor outcome without heart transplantation^[^^2^^]^. The clinical presentation of GCM is usually dramatic, but some may present an indolent course with the presence of symptoms for months to years before the proper diagnosis is made^[^^3^^]^. The natural course is rapid and mortality is high if left untreated, with an average transplant-free survival of <6 months. Ventricular assist device placement and immunosuppressive regimens, including high dose steroids and cyclosporine have modestly improved the prognosis of GCM, to almost 12 months of transplant-free survival; nevertheless, they still need heart transplantation^[^^2^^]^. In 2013, Larsen et al.^[^^1^^]^, stated that GCM located in the atrium have distinctive clinicopathologic features with a more favorable prognosis than classical ventricular GCM. After the first description of atrial GCM in 1964, the largest series of atrial GCM was published by Larsen et al.^[^^1^^]^ with 6 consecutive patients and in the literature only 13 cases were found in the PubMed database search. After Larsen called attention to distinct features of atrial GCM, Basso and Thiene^[^^4^^]^ stated that atrial GCM might be more common than generally believed. As reviewed, 5 of these literature based cases lacked history or evidence of rheumatic diseases, as did in our case. Our case also emphasizes that atrial GCM is a distinctive disorder with its own characteristics. As Basso and Thiene^[^^4^^]^ stated, the disease might be attributable to atrium-specific autoantigens.

Tumors of the cardiovascular system are very rare diseases. There are many disorders that do not fit into the concept of tumor or neoplasm. Among these cardiovascular tumors, 70% of these are benign, and when surgically excised, most of them are pathologically confirmed as benign^[^^5^^]^. Tumors of the heart are divided into three categories: benign tumors and tumor-like lesions, malignant tumors, and pericardial tumors. In benign tumors, tumors were classified as tumors showing differentiation into muscle cells such as rhabdomyoma, adult cellular rhabdomyoma, hamartoma of mature cardiac myocytes, and histiocytoid cardiomyopathy. Cardiac myxoma and papillary fibroelastoma are classified as pluripotent mesenchymal origin, and cardiac fibroma and inflammatory myofibroblastic tumor were classified as tumor showing differentiation into myofibroblastic cell.

An atrial myxoma must be differentiated from a LA thrombus. Echocardiographically and surgically, the presence of a stalk and mobility favors atrial myxoma whereas the thrombus is usually situated in the posterior portion of the atrium with a layered appearance. On the other hand, myocarditis is a diffuse inflammatory process of the myocardium, although instances of focal myocarditis in the right/LV or LA may be present rarely as in our case.

Rheumatic fever (RF), sarcoidosis, specific infections and gout should be considered in the differential diagnosis. Both GCM and cardiac sarcoidosis (CS) are presented in similarly aged patients. Unlike GCM, CS is characterized by tight nonnecrotizing granulomas, with central multinucleated giant cells. Fibrosis is more pronounced in CS than in GCM^[^^6^^]^. The absence of necrosis of rheumatoid myocarditis without Aschoff cells excluded the diagnosis of RF and the absence of granulomatous structures in our case excludes sarcoidosis in the differential diagnosis. Both GCM and CS may present with ventricular tachycardia, presentation with acute-onset heart failure is more commonly seen in GCM, while presentation with heart block is more predictive of CS^[^^6^^]^. Although GCM does not form a mass lesion in general, it is not a must and this is the first report of atrial GCM forming a mass lesion in the literature.

We believe that more case reports and possibly series will be reported in near future as the importance and unique properties of this disease are learned. In this case, we clearly identified the borders of the mass limited in the LA and total removal of the mass improved patient's quality of life with no additional treatment.

**Table:** 

Authors' roles & responsibilities	
OT	Substantial contributions to the conception or design of the work; or the acquisition, analysis, or interpretation of data for the work; drafting the work or revising it critically for important intellectual content; final approval of the version to be published
YD	Substantial contributions to the conception or design of the work; or the acquisition, analysis, or interpretation of data for the work; drafting the work or revising it critically for important intellectual content; final approval of the version to be published
NG	Substantial contributions to the conception or design of the work; or the acquisition, analysis, or interpretation of data for the work; drafting the work or revising it critically for important intellectual content; final approval of the version to be published
MCA	Substantial contributions to the conception or design of the work; or the acquisition, analysis, or interpretation of data for the work; drafting the work or revising it critically for important intellectual content; final approval of the version to be published
